# Does Acute Beetroot Juice Supplementation Improve Neuromuscular Performance and Match Activity in Young Basketball Players? A Randomized, Placebo-Controlled Study

**DOI:** 10.3390/nu12010188

**Published:** 2020-01-09

**Authors:** Álvaro López-Samanes, Aarón Gómez Parra, Victor Moreno-Pérez, Javier Courel-Ibáñez

**Affiliations:** 1 School of Physiotherapy, Faculty of Health Sciences, Universidad Francisco de Vitoria, 28223 Madrid, Spain; alvaro.lopez@ufv.es; 2 Faculty of Sport Sciences, University of Murcia, 30720 Murcia, Spain; deporte.salud18@gmail.com; 3 Center for Translational Research in Physiotherapy, Department of Pathology and Surgery, Universidad Miguel Hernández, Elche, 03550 San Juan, Spain; vmoreno@goumh.umh.es

**Keywords:** nitric oxide, match demands, GPS, team sports, ergogenic aid

## Abstract

Whereas beetroot juice (BJ) supplementation is shown to increase physical performance in endurance activities, its benefits in team sports has been barely studied. In this randomized placebo-controlled study, we investigated the effects of BJ acute supplementation in improving neuromuscular performance and physical match activity in basketball. Ten young male competitive basketball players aged 15–16 years received 140 mL of BJ or placebo (PLA) on two separated days in a balanced cross-over design. Testing sessions comprised a neuromuscular test battery consisting of a countermovement jump (CMJ), isometric handgrip strength, 10-m/20-m sprint and agility T-test, followed by a 40-minute simulated basketball match. Physical match activity (distances, speeds, accelerations, and decelerations) was monitored using an inertial tracking system (Wimu Pro^TM^) Results revealed no significant effects of BJ on CMJ (*p* = 0.304, ES = 0.13), isometric handgrip strength (*p* = 0.777, ES = 0.06), 10-m (*p* = 0.820, ES = 0.10), and 20-m sprint (*p* = 0.540, ES = 0.13), agility T-test (*p* = 0.979, ES ≤ 0.01) and any physical match demands (*p* > 0.151, ES = 0.13–0.48). Acute moderate doses of BJ (12.8 mmol of NO_3_^−^) was not effective in improving neuromuscular performance (jump height, isometric handgrip strength, sprint, and agility) or physical match requirements in young trained basketball players the day of the competition.

## 1. Introduction

Team sports competitions like basketball are becoming increasingly intense, characterized by stronger athletes, explosive demands, and repeated high-intensity intermittent efforts at early stages [1,2]. The use of nutritional supplements and ergogenic aids to directly or indirectly enhance performance is increasing in popularity among stop-and-go intermittent exercise [3], but only a few have good evidence of benefits [4,5], such as caffeine, creatine, sodium bicarbonate, beta-alanine, and nitrate (NO_3_^−^). While previous studies in basketball have tested the ergogenic effects of caffeine [6,7,8], creatine [9], and sodium bicarbonate [10], the efficacy of NO_3_^−^ supplementation to positively impact physiology and performance is still unknown. 

Dietary NO_3_^−^ supplementation though beetroot juice (BJ) has experienced a boom since 2009 and it is nowadays one of the most popular ergogenic aids in endurance training [11] and health-related exercise [12]. BJ is rich in NO_3_^−^, a precursor of nitric oxide (NO) through the nitrate–nitrate–NO pathway [13]. NO_3_^−^ supplementation improves vasodilation and increases blood flow in muscle [14,15] that appears to benefit muscle force and power production in rodents [16] and healthy adults [17]. After consuming BJ, NO_3_^−^ reduction elicits increases in plasma nitrite (NO_2_^−^) which serves as a substrate for O_2_- independent NO synthesis and favours the reduction of NO from NO_2_- circulating in plasma under conditions of hypoxia and acidosis [18]. Furthermore, NO_3_^−^ has been suggested may be particularly effective at augmenting different physiological processes such as calcium handling proteins and contractile force in type II (fast-twitch) muscle fibers [19]. Due to these benefits might positively impact on physical performance during intermittent efforts, repeated sprinting bouts, jumps, or changes of direction, the interest in BJ supplementation in team sports like soccer [20,21], rugby [21,22], hockey [21,22], or water polo [23] is increasing lately. Nonetheless, in contrast to the well-established effectiveness of dietary BJ supplementation in endurance athletics events [4,5], more research is required to determine its potential to increase physical performance during a team-sport competition [24]. 

The first approach in examining the effects of BJ in team sport-specific intense intermittent exercise performance [25] found increments around 3.5% in Yo–Yo IR1 test in recreational team-sport players after consuming 490 mL of concentrated, nitrate-rich BJ. These improvements in the Yo–Yo IR1 have been reproduced in soccer players [20] and team-sport athletes including hockey, soccer, and rugby teams [21] after 5 and 6 days of 140 mL/day, respectively. Additionally, this 1-week supplementation was found to produce changes in sprint times [21] and total work after a prolonged an intermittent sprint test [22]. These improvements however have been equivocal in similar studies [26] and even questioned due to the little magnitude of change (< 3%), which might not be likely to produce large effect in performance throughout a team-sport play [27]. Likewise, the effects of acute BJ supplementation in neuromuscular functional performance like jumping capacity has been scarcely investigated and with controversial results [28,29]. Furthermore, there are no studies on the effects of BJ on change of direction speed, despite being considered an essential ability for success in team sports [30,31,32].

In addition, the effects of BJ supplementation in improving physical performance and match running load or real exposure during competition (i.e., total distance covered, speed achieved, number of accelerations and decelerations) in team sports remains unknown. Thanks to advances in microtechnology and the development of tracking, wearable sensors such as global positioning systems (GPS), it is possible to quantify the external load during competitive matches [33,34]. Nevertheless, there is no previous study using this technology in team-sport players to quantify changes in activity performance during competition after BJ supplementation.

Therefore, to increase knowledge on the potential ergogenic effects of nitrate-rich compound supplementation in intermittent effort and team sports, we conducted a randomized, double-blinded placebo-controlled, crossover trial, to identify changes in neuromuscular performance and physical activity requirements during match competition on trained young basketball players produced after moderate acute doses of BJ. 

## 2. Materials and Methods 

### 2.1. Participants

Young basketball players were recruited meeting the following inclusion criteria: (i) having between 15 and 16 years old; (ii) more than five years of basketball training experience; (iii) no contraindications to BJ diet; and (iv) no physical limitations, health problems, or musculoskeletal injuries. Twelve competitive male basketball players from the same regional club, aged 15.6 ± 0.5 years, body weight 76.3 ± 9.0 kg, height 184.3 ± 7.5 cm, body mass index 22.5 ± 2.9, each with more than eight years of basketball training experience (2–3 sessions a week, 1–2 h per session), were screened and recruited as potential participants. After being fully informed of the experimental protocols, all players gave their informed and parental written consent to participate. Ten players (four guards, four forwards, and two centers) were selected as the study sample while the resting two were considered if someone drops out of the study to ensure a 5-on-5 basketball competition. All the participants were familiarized with the testing procedures as part of their pre-season assessment The Bioethics Commission of the University of Murcia (ID: 2421/2019) approved the study which complied with the recommendations of the Declaration of Helsinki. 

### 2.2. Experimental Design

**Figure 1:**
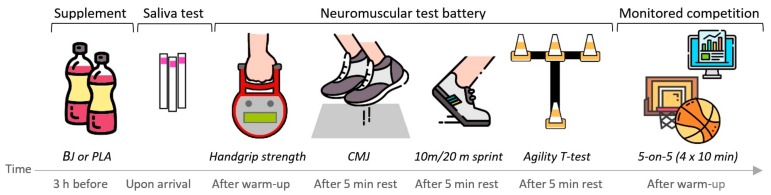
Experimental design of the study.

The study design was randomized double-blinded and placebo-controlled crossover trial (NCT04210531). Basketball players completed two identical testing sessions in two different days with one week between to allow a full recovery and substance wash out. Participants were allocated to receive a 140 mL dose of BJ or a masked placebo (PLA) 3 h before each testing session [20] according BJ dietary supplementation recommendations [35]. Prior to the evaluations, players underwent two familiarization sessions including body composition assessment, the neuromuscular battery test, and a 10-minute 5-on-5 game wearing the GPS. Testing sessions included a neuromuscular test battery consisted in countermovement jump (CMJ), isometric handgrip strength, a modified version of the agility T-test, and 10-m and 20-m sprint tests, followed by a 40-minute monitored basketball match (Figure 1). Experimental procedures were performed at the same hour in the evening (19:00 h) to avoid the influence of circadian rhythms on performance such as previously reported in other intermittent sports [36]. Environmental conditions were measured using a portable weather station (WMR 108, Mextech, India) and the Windy app for Android, with all the measurement made under similar conditions (16 °C, 45% humidity, wind < 5 km·h^−1^). All testing sessions started with a saliva test to verify that the supplement was taken and a 15-minute standardized dynamic warm-protocol, consisting of 5 minutes of jogging, 5 minutes of joint mobility, 2 × 15 m progressive accelerations with 1 minute of rest between, 5 progressive jumps, and 1 practice trial for each test. 

### 2.3. Sample Size, Randomization, and Allocation 

**Figure 2:**
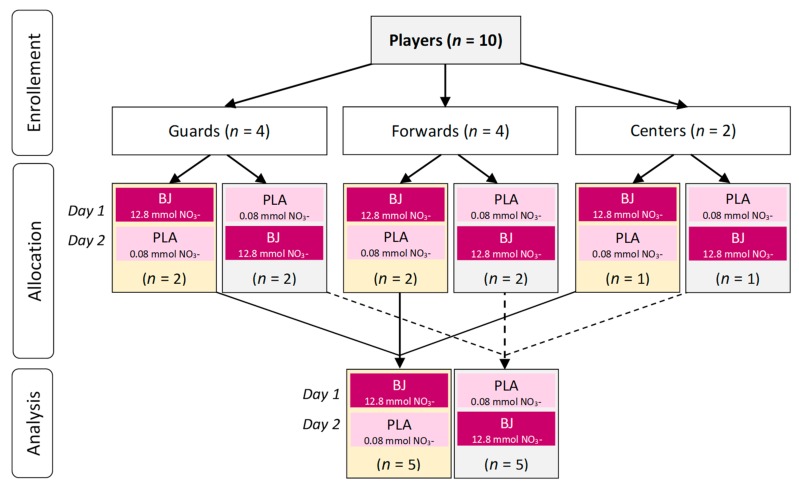
Participant allocation according to beetroot juice (BJ) or placebo (PLA) conditions.

The required sample size was determined by statistic power calculation on the basis of previous studies [37]. The minimum number of participants required to detect an 8 ± 6 % difference in counter movement jump (CMJ) performance between two groups, with a power of 0.80 and two-tailed α level set at 0.05, was estimated as seven per group using the sample size package for R (v. 3.6.1). One researcher (A.L.S.) allocated all the participants’ drinks in a randomized crossover design (on each testing session, 50% of participants ingested PLA and 50% ingested BJ beverages) using the Research Randomizer (www.randomizer.org) and considering players specific position (i.e., guards, forwards, and centers) (Figure 2). Team members for the basketball match were drawn including two guards, two forwards, and one center on each team. Both teams included players from each experimental condition (BJ and PLA) according to the randomization.

### 2.4. Nutritional Intervention

Three hours before initiating the neuromuscular test battery, participants were provided with one serving of either 140 mL of BJ (12.8 mmol of NO_3_^−^; Beet-It-Pro Elite Shot, James White Drinks Ltd., Ipswich, UK) or PLA drink (0.08 mmol of NO_3_^−^; Salud Viva, Nano Salud, Alicante, Spain), as described elsewhere [38]. All participants were instructed to follow a diet sheet the day before each testing session, consisting of 60% carbohydrates, 30% fat, and 10% proteins. Dietary NO_3−_ was limited by providing subjects a list of NO_3−_ rich foods (e.g., beetroot, celery, or spinach) that they should avoid in the 48 h before each testing session. Additionally, in the 24 h leading up to each session, subjects were encouraged to avoid brushing their teeth; using an oral antiseptic rinse; or ingesting gum, sweets, or stimulants (e.g., caffeine) that could alter the oral microbiota and interfere with NO_3_^−^ reduction. Saliva test strips (Nitric Oxide Saliva Test Strips, Berkeley Life, Chicago, USA) were used upon arrival to the testing site to verify that the supplement was consumed, as per the manufacturer’s guidelines and previous studies [39].

### 2.5. Neuromuscular Battery Tests

Isometric handgrip strength was measured twice for the shooting (dominant) hand using a dynamometer (Takei 5101, Tokyo, Japan), with 30 s of rest [40]. Maximal voluntary contraction (MVC) was recorded in kg, adjusted to each subject’s body weight and converted to newtons by multiplying the kg value by 9.8 [41]. Following 5 minutes of recovery, participants completed three CMJ, with their hands on the hips, separated by 45 s rest. Maximal vertical jump height was determined using a contact platform (ChronoJump Boscosystem v. 1.9.0, Barcelona, Spain). Players performed two maximal 20-m sprints, separated by a 3-minute rest. Sprint times were measured using three double-beam photocell timing gates (ChronoJump Boscosystem v. 1.9.0, Barcelona, Spain), placed at 0, 10, and 20-m, so that the times to cover 0–10-m and 0–20-m were determined. Each sprint was initiated from a standing position, 1-m behind the photocell gate, which started a digital timer. Finally, players completed two trials of the modified agility T-test with 2-minute rest between [42], consisting of five sprints as follows: 5 m forward, side-shuffle 2.5 m to the left, side-shuffle 5 m to the right, side-shuffle 2.5 m to the left, and 5 m backwards until crossing the starting line. Two double-beam photocell timing gates (ChronoJump Boscosystem v. 1.9.0, Barcelona, Spain) were set 1-m above the surface and positioned 3-m apart facing each other on either side of the starting/finishing line. Participants began each test 1 m behind the starting line, and the timer started when they passed the first gate. All the tests were performed in the basketball court. The best performance for each test was considered for the analysis. Measurements were obtained using devices with very high reliability [43,44,45] and according to standard procedures with high intraclass-correlation (ICC > 0.903) and low coefficient of variation (CV < 1.5 %) [36,46]. All these tests has been successfully used for physical performance assessment in young basketball players [47,48,49].

### 2.6. Basketball Game Activity

Players competed in a 5-on-5 basketball match on an official outdoor court, regulated by two referees and following the FIBA rules. The game consisted of four parts of 10 minutes with a break of 2 minutes between them. No substitutions were allowed to avoid data inconsistency. Each player was monitored using a 10-Hz portable GPS and accelerometer units (Wimu Pro^TM^, RealTrack Systems, Spain). These devices has shown a good level of accuracy for assessing the distance covered [50]. According to the manufacturer’s recommendations, all devices were activated 15-minutes before data collection to allow acquisition of satellite signals and synchronization of the GPS clock with the satellite’s atomic clock. Following match-play competition, data were downloaded to a personal computer and analyzed using the system-specific software (SPRO Software, Realtrack Systems SL, Spain). We used the following variables to assess the external load during match-play, total distance covered per minute [51] (walking (< 6.0 km·h^−1^), jogging (6.0 to 12.0 km·h^−1^), running (12.1 to 18.0 km·h^−1^) and high-intensity running (18.1 to > 24.0 km·h^−1^)), the number of high intensity accelerations (> 2m∙s^−2^) decelerations (< -2m∙s^−2^), player load and peak velocity according to the literature [34,52] during the 40 minutes match. The match intensity was assessed by means of heart rate mean (HR_mean_) and maximum (HR_max_) parameters and the 10-point rate of perceived exertion scale (RPE) was obtained 30 minutes after of the end of the match [53] 

### 2.7. Statistical Analysis

Data are presented as means and standard deviation (M ± SD), percentage of change (%) and 95% confidence interval for the mean difference (95% CI M_diff_). The Shapiro–Wilk test was used to verify the assumption of normality. The homogeneity of variance across groups (BJ and PLA) was checked using the Levene’s test. Repeated measures ANCOVA was used to examine the effect of the BJ nutritional intervention in neuromuscular performance and game activity parameters, considering players’ specific position as a between-subjects factor. When appropriate, post hoc comparisons were accomplished via Scheffé’s test. The significance level was set at *p* < 0.05. Cohen’s *d*_av_ was calculated to estimate the effect size [54], considering trivial (< 0.19), small (0.20–0.49), medium (0.50–0.79), and large (> 0.80). A common language (CL) effect size was also calculated to provide a more intuitive metric, interpreted as the probability (%) that a person scores higher on one mean compared to the other, after controlling for individual differences [55]. Statistical analyses were performed using an excel spreadsheet [54] and the SPSS version 22.0 (IBM Corp., Armonk, NY, USA). Figures were designed using GraphPad Prism 6.0 (GraphPad Software Inc., San Diego, California, USA).

## 3. Results

One participant was unable to attend the scheduled second testing sessions due to vehicle problems, therefore data from the nine participants who completed the experiment were considered for the analysis. The study blinding was successful with 56% of the participants (5/9 participants) correctly identifying the supplement that they were receiving. The 100% of participants were compliant with the supplementation. The nutritional strategy was well tolerated without severe adverse effects, and only one player showing discomfort after the PLA ingestion.

**Figure 3:**
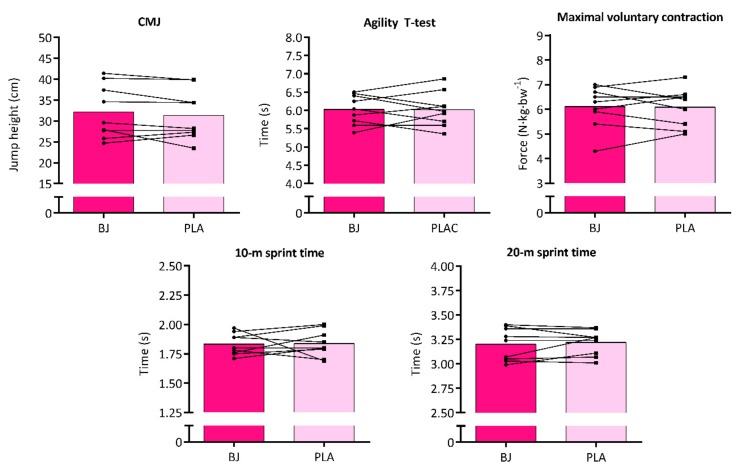
Neuromuscular battery test results for beetroot juice (BJ) and placebo (PLA) conditions.

Results from the neuromuscular battery tests comparing BJ vs. PLA conditions are shown in Figure 3, with no significant effect of the nutritional intervention in any case: CMJ height was 2.5% higher (95% CI M_diff_ = -0.7 to 2.4 cm; *p* = 0.304; *d*_av_ = 0.13; CL effect size = 66%), 10-m sprint was 0.2% faster (95% CI M_diff_ = -0.1 to 0.1 s; *p* = 0.820; *d*_av_ = 0.10; CL effect size = 53%), and 20-m sprint was 0.6% faster (95% CI M_diff_ ≤ -0.1 to 0.1 s; *p* = 0.540; *d*_av_ = 0.13; CL effect size = 58%) and MVC for isometric handgrip increased 0.1% (95% CI M_diff_ = -0.3 to 0.4 N·kg·bw^−1^; *p* = 0.777, *d*_av_ = 0.06; CL effect size = 54%), whilst the agility T-test time minimally increased 0.1% (95% CI M_diff_ = -0.3 to 0.3 s; *p* = 0.979, *d*_av_ ≤ 0.01; CL effect size = 50%). No within- or between-subjects’ main effects or interactions were found in any neuromuscular tests.

The level of competition for the two matches were similar (final score difference < 10 points) with no statistical difference in any intensity parameters (RPE = 4.8 ± 1.2 and 4.7 ± 1.4; *p* = 0.865; HR_max_ = 188 ± 12 vs. 192 ± 9 bpm, *p* = 0.200; HR_mean_ = 156 ± 44 and 159 ± 11 bpm, *p* = 0.332). Players’ activity during the basketball match-play showed no significant changes regarding the supplementation condition in any of the physical demands (Table 1) or HR parameters measured (HR_max_ = 185 ± 16 vs. 192 ± 9 bpm, *p* = 0.154; HR_mean_ = 156 ± 14 vs. 158 ± 11 bpm, *p* = 0.179). No within- or between-subjects’ main effects or interactions were found in any game activity parameter.

## 4. Discussion

This study showed for the first time that BJ acute supplementation did not produce any statistically significant improvement in neuromuscular or physical activity performance during match-play in young trained basketball players. Trivial changes were observed in jump height, sprint times and handgrip strength in favour to BJ, but did not reaching statistical significance in any case. Likewise, players showed trivial to small changes in a number of physical activity parameters after BJ ingestion, but not high enough to be considered to improve players’ performance during match in young trained players. These findings add to the limited literature on the ergogenic effects of nitrate-rich compound supplementation like BJ in team sports and intermittent effort. Additionally, we provide new evidence on the limited effect of acute BJ ingestion in improving players’ physical activity demands during a 5-on-5 basketball game using an inertial tracking system. 

After acute (3 h before testing) moderate dose (12.8 mmol of NO_3_^−^) of BJ supplementation, trained basketball players showed small performance variations in CMJ maximum height (2.5%), handgrip MVC (0.6%), 10-m (-0.2%) and 20-m sprint times (-0.6%), and agility (-0.1%) without statistical relevance. These findings indicate that 140 mL of acute BJ ingestion did not improve neuromuscular performance in young trained basketball players, at least in jumping, isometric handgrip strength, sprint, and agility values. Our results support those reported in healthy resistance-trained men [29], who showed low and non-significant improvements in the CMJ height (2.3%, *p* = 0.863) after consuming a low dose of BJ (6.4 mmoL of NO_3_^−^) compared to placebo. Likewise, acute BJ supplementation seems to be not enough to produce increments in handgrip strength, in contrast to what was observed after chronic administration [41]. One previous study found significant but limited improvements in sprint times (-1.2% and -1.6% for 20-m and 10-m respectively) after 5 days of BJ (6.4 mmoL of NO_3_^−^) supplementation [21]. However, these results have not been totally supported by previous studies [26] and considered meaningful in the real competition [27]. Although we did not find any differences regarding the players’ specific position, it might be arguable that individual physical characteristics may influence their improvements. Particularly in team sports like basketball, players have well-defined roles that requires specific and distinctive physical attributes [32,33,56]; hence, a different response to BJ supplementation can be expected. In this sense, it can be arguable that athletic and explosive players (e.g., guards or forwards) would have greater benefits. However, whether individual team-sport athletes respond different to BJ supplementation is unknown. 

Whereas it seems clear that NO_3_^−^ improves muscle efficiency and recovery after severe-intensity, constant work-rate exercise [13], its benefits on delaying fatigue after intermittent, high-intensity exercise efforts remains unclear [24]. Furthermore, despite dietary nitrate appears to be effective in increasing muscle speed and power for health purposes [57], acute doses of NO_3_^−^ may not be enough to cause improvements on contractile properties of the fast-twitch muscle during high-intensity and short-duration efforts (i.e., jumping or sprinting) in young trained athletes [26,58]. This may happen due to insufficient activation of the soluble guanyl cyclase-cyclic guanosine monophosphate-protein kinase G [16,59], which seems to be the pathway by which NO modulates skeletal muscle contraction [60]; nonetheless, it remains to be demonstrated whereas an increase in NO bioavailability as a result of dietary NO_3_^−^ intake may elicit these mechanisms [60]. Thus, more research is needed to confirm animal-based findings and determine the possible effects of BJ supplementation on neuromuscular performance in team sport athletes. Likewise, future studies should investigate whether or not a balanced NO_3_^−^−rich diet might produce comparable effects in physical performance to those observed after acute or chronic supplementation.

A novel contribution of our study is the use of inertial tracking systems to determine changes in players’ physical activity throughout a team-sport play after BJ supplementation. Inertial-based wearable technology is increasing in popularity among automatic data collection tools in team sports, due to the list of parameter that coaches and researchers can automatically obtained for each player during a training session or a match-play competition [61,62]. Previous studies have used this technology to determine the physiological demands in young basketball players [34] obtaining a similar physical match activity than our study. We found a not significant effect of acute BJ supplementation on any external load parameter throughout the match. These results suggest that nitrate-rich compound ingestion could not be an effective aid to increase physical performance on the day of the competition. 

This work has some important strengths such as the use of inertial tracking systems and high-quality testing equipment. Furthermore, this appears to be the first study quantifying the effects of BJ supplementation throughout a team-sport play. There are also some limitations that should be considered when interpreting the results. The sample size is reduced and the inclusion of a small number of participants from all positions meant it was not possible to determine differences between players’ specific position and match-play related measures. Future studies should consider adults, professionals, or elite players to confirm our findings. In addition, the extent to which team sport athletes with different physical attributes, playing positions, and characteristics can respond to nitrate-rich compound ingestion is an open challenge.

## 5. Conclusions

The ingestion of acute moderate doses of BJ (12.8 mmoL of NO_3_-) the day of the competition did not improve neuromuscular performance (jump height, isometric handgrip strength, agility, and sprint) or physical match-play activity (total distanced covered, number of high intensity accelerations and decelerations, player load, and peak velocity) compared to placebo, in young trained basketball players.

## Figures and Tables

**Table 1:** External load values (means ± SD) obtained in placebo and beetroot conditions during basketball match-play

Variables	Placebo	Beetroot Juice	*Diff*	*p*	*d*_av_
Relative distance (m·min^−1^)	67.99 ± 5.39	69.14 ± 8.20	3.3%	0.722	0.21
Peak Speed (km·h^−1^)	22.18 ± 1.62	22.86 ± 2.27	2.8%	0.441	0.34
< 6 km·h^−1^ (m·min^−1^)	31.97 ± 2.69	31.00 ± 3.31	-2.0%	0.513	0.32
6.0 to 12.0 km·h^−1^ (m·min^−1^)	24.27 ± 2.40	25.62 ± 3.18	5.1%	0.243	0.48
12.1 to 18.0 km·h^−1^ (m·min^−1^)	9.92 ± 2.16	10.53 ± 2.15	6.5%	0.404	0.28
18.1 to > 24.0 km·h^−1^ (m·min^−1^)	1.44 ± 0.86	1.28 ± 0.59	-4.4%	0.539	0.22
PlayerLoad (AU)	1.05 ± 0.13	1.07 ± 0.17	2.0%	0.565	0.13
Total accelerations (*n*)	17.36 ± 0.92	17.71 ± 0.62	2.1%	0.151	0.44
Total decelerations (*n*)	16.88 ± 0.94	17.22 ±0.76	1.2%	0.715	0.39
Accelerations > 2 m·s^−2^ (m·min^−1^)	1.86 ± 0.34	1.95 ± 0.49	3.0%	0.546	0.21
Decelerations < 2 m·s^−2^ (m·min^−1^)	1.67 ± 0.50	1.78 ± 0.45	8.5%	0.376	0.23

Abbreviations: AU = arbitrary units; *d*_av_= Effect size mean; m = meters; min = minute; *n* = number
